# What determines psychological well-being among Iranian female adolescents? Perceived stress may overshadow all determinants

**DOI:** 10.15171/hpp.2018.10

**Published:** 2018-01-07

**Authors:** Haleh Hezomi, Haidar Nadrian

**Affiliations:** ^1^Department of Health Education & Promotion, Faculty of Health, Tabriz University of Medical Sciences, Tabriz, Iran

**Keywords:** Psychological wellbeing, Perceived Stress, Adolescents, Female

## Abstract

**Background:** Mental health problems, as one of the most neglected issues among adolescents,are common during adolescence and emerging adulthood. The aim of present study was to investigate the determinants of psychological well being among female adolescents in Tabriz,Iran.

**Methods:** In this cross-sectional study, multi-stage cluster sampling was employed to recruit 289 female high school students to participate in the study during 2013–2014. A 3-section questionnaire was applied to collect data. Hierarchical linear regression analysis was applied to illustrate the variations in psychological wellbeing score on the basis of socio-demographic and psychological variables.

**Results:** Self-efficacy, hopefulness, happiness and life satisfaction were positively correlated (r> 0.400) and perceived stress was negatively associated with psychological well-being (r =-0.689). In the first model, satisfaction with family lifestyle (β = 0.168, P < 0.001) and perceived stress (β = -0.470, P < 0.001) were the most significant positive and negative predictors for psychological wellbeing, respectively (R^2^ = 0.595, P < 0.001). In the second model (step 6),physical activity (β = -0.109, P < 0.019), have/had boyfriend (β = 0.237, P < 0.001), hopefulness(β = -0.130, P < 0.05) and happiness (-β = 0.387, P < 0.001) were significant predictors for perceived stress (R^2^ = 0.453, P < 0.001).

**Conclusion:** Considering the various behavioral, mental and social predictors of psychological wellbeing, it seems that perceived stress has overshadowed the influence of a majority of the other factors. Such influence may be due to the specific cultural and context-based rules enforced for female adolescents in the Iranian community.

## Introduction

Mental health problems, as one of the most neglected issues among adolescents,^1^ are common during adolescence and emerging adulthood.^2^ The prevalence rate of major depressive disorders in Iran is reported to be 4.1%.^3^ Previous studies in Iran have reported the prevalence of mental disorders to be from 11.9% to 23.8%.^4,5^ Globally, mental disorders and mental health problems seem to have been considerably increased among adolescents in the past decades. It is also a growing public health concern among children and adolescents of developing countries.^6,7^ An increase in self-reported stress-related and mental health problems among girls and young women is shown in several nationwide surveys. A systematic review on Iranian studies has shown a wide range for the prevalence rates of mental disorders among high school students and has thus suggested more studies with improved quality in this field.^8^

Among the adolescents, mental health may be related to health behaviors such as smoking, alcohol use and physical activity. Female adolescents are in particular need for effective programs to develop healthy lifestyle behaviors.^9^ Thus, it is important to consider both health behavior and mental health while exploring the independent contributions of these factors. In the domain of healthy lifestyle and prudent health behaviors, previous studies have reported significant associations between happiness and regular physical activity^10^ or higher levels of physical activity,^11^ not smoking or less cigarette use^12^ and healthy diet.^12^ Therefore, there is less likelihood for happy individuals to engage in damaging and unhealthy behaviors.

Among adolescents, academic pressures and social isolation may be risk factors for the development of mental health problems,^13^ as well. To maintain their mental health, friendship is particularly important.^14^ The results of studies performed on the importance of friendships for mental health among college students, the styles of teenagers’ relations, and the formation of friendships during interpersonal stress, have indicated that friendship and interpersonal relations are very important to protect mental health among adolescents.^15^

A previous study in Iran reported that 61.7% of the adolescent female students in Tehran had some degree of anxiety.^16^ In another Iranian study, a majority of the students was reported to be with mental health problems.^17^ In developing countries, there is a lack of research on the self-reported wellbeing among female adolescents.^18^ Also, few researches have focused on comparing subjective wellbeing among different adolescent groups (e.g. age, economic status, ethnicity, parental education).^19^ On the other hand, few studies in developing countries have considered how perception on facts in daily living (such as perception on the economic status of family, religion, and healthy lifestyles) among female adolescents may be associated with psychological wellbeing at the presence of psychological constructs (like happiness, hopefulness and stress). Thus, the aim of the present study was to investigate the determinants of psychological wellbeing among female adolescents in Tabriz, Iran. The following questions guided our study: (1) How is psychological wellbeing among female adolescents in a developing country, like Iran?, (2) How the Iranian female adolescents perceive the facts of daily living?, and (3) Does perceptions on facts in daily living predict psychological wellbeing, at the presence of psychological constructs (like happiness, perceived self-efficacy and stress)?

## Materials and Methods

### Participants and procedure

In this cross-sectional study, multi-stage random sampling was employed to recruit 289 female high school students (in the ninth grade of education) in Tabriz, northwest of Iran, to participate in the study during 2013–2014. Among the 5 educational districts in Tabriz, one district was randomly selected, from which 2 high schools with similar local characteristics (i.e., geographic location) and educational curriculums were selected for final recruitment. Quota sampling method was used to elect the samples from each class. Written permission was obtained from the students and their parents before the study initiation, and all signed consent forms. Also, instructions to complete the study questionnaire were provided for the respondents. All the high school female students studying in the selected high schools were included. The students with mental limitations, based on their health status records in the data registry of the schools were excluded. Also, the students who were under psychiatric treatment and those who were reluctant to participate in the study were excluded.

### Measures

*Demographic characteristics* of the students included the following 7 items: age (14/15), number of family members (3/4/≥5**)**, grade of birth (1/2/>3), father’s job (self-employed/employee), mother’s job (housewife/employee), father’s education (elementary/ diploma/university), mother’s education (elementary/diploma/university), economic status of family (moderate/good/very good), and having religious beliefs (low/moderate/high/very high).

*Perception of the students on some facts in daily living* were assessed using the following 9 items: being satisfied with the parent’s job (Yes/No), being satisfied with family lifestyle (Yes/No), belief on chance in daily living (Yes/No), willingness to create daily happy times for oneself (like listening to music/watching movie with friends/family) (Yes/No), willingness to watch TV (Yes/No), willingness to daily listening to music (Yes/No), commitment to healthy action plans in daily life (like routine tooth brushing/physical activity) (Yes/No) and take care about health (Yes/No).

*Perception on social relationship* in daily life was investigated using a 3-item researcher-made scale, including the following items: having favorite social relations with friends/relatives, willingness to have social relation with friends/relatives, have/had boyfriend. A Yes = 1/No = 0 response format was considered as answer choices for the items. The scale score ranged from 0-3, in which the higher score indicated a better level of social relationship in daily life.

*Healthy lifestyle* was assessed applying another 3-item researcher-made scale. The items were as follows: having the history of smoking (cigarette/hookah) in the lifelong, having a healthy diet (like eating more fruits/vegetables, less fast foods, and less fatty foods in a day), having at least 30 minutes of moderate physical activity (like walking) per day. A Yes = 1/No = 0 format was considered as answers to the items. The scale score ranged from 0-3, in which the higher score indicated a healthier lifestyle.

*General Health Questionnaire:* The Persian version^20^ of General Health Questionnaire (GHQ-28) was used to assess psychological well-being of the respondents. This instrument includes 28 items and 4 subscales. Each subscale of the questionnaire includes 7 items in the somatic symptoms, anxiety/insomnia, social dysfunction and severe depression domains. All items of the scale are scored on a 4-point scale (0–3) and a higher score indicated poorer mental health status. The estimated alpha coefficient for the translated version of the questionnaire was reported to be 0.93. The lowest and the highest possible scores were 0 and 85, respectively. A higher score indicates poorer mental health status.

*Happiness Questionnaire:* The Persian version^21^ of the Oxford Happiness Questionnaire was used to measure happiness. This questionnaire comprises 29 items with response format based on a 6 points Likert-type scaling from “strongly disagree” to strongly agree”. The lowest and the highest possible scores could be 1 and 6. The lowest and the highest possible scores were 29 and 174, respectively. Higher scores on the scale represent higher levels of happiness. The reported estimate of the alpha coefficient for the translated version of the questionnaire was 0.90 indicating internal consistency of the scale.

*Self-efficacy Questionnaire:* The Persian version^22^ of the Sherer’s general self-efficacy scale was used to measure general self-efficacy. Each item in the scale was measured applying a 5-point Likert type scaling ranged from “strongly disagree” (1) to “strongly agree” (5). The lowest and the highest possible scores were 17 and 85, respectively. Validation of this questionnaire in Iran was carried out by Charkhabi et al^22^ and the reported alpha coefficient was 0.83.

*Stress Questionnaire:* Perceived stress was measured using the Persian validated version^23^ of the Cohen’s Perceived Stress Scale. The scale consists of 10 items based on a 5-point Likert type scale ranging from 0 (never) to 4 (very often). The lowest and the highest possible scores were 0 and 40, respectively. A higher score in the scale represents the lower level of stress.^24^

*Hopefulness Questionnaire:* Hopefulness was measured by the Persian version^25^ of the Snyder’s Hopefulness Scale^26^ which includes 12 items with a 4-points Likert-type scale ranging from 1 (deﬁnitely false) to 4 (deﬁnitely true). The lowest possible score was 12 and the highest was 48. The reported estimated alpha coefficient for the translated version of the questionnaire was 0.67.

*Satisfaction of Life Questionnaire:* The Persian version^27^ of Satisfaction with Life Scale (SWLS) was applied to measure life satisfaction which was originally developed by Diener et al.^28^ This scale has 5 items. An example of the items is as follows: ‘‘in most ways my life is close to my idea1.” Responses to the items are coded based on a 7-point Likert type scale from strongly disagree (1) to strongly agree (7). The lowest possible score was 5 and the highest was 35. The reported estimated reliability coefficient for the Persian translated version of the questionnaire was 0.82, indicating a reliable internal consistency.

### Statistical analysis 

All the data analysis was performed in SPSS version 16 for Windows. Data were presented by mean (SD) and frequency (%) for quantitative and qualitative variables, respectively. The normality of quantitative variables was assessed and confirmed by one-sample Kolmogorov–Smirnov (K-S) test. Summary statistics and frequency distributions were used to describe and interpret the meaning of data. An additional calculation was performed on the mean score of the variables. As the mean and standard deviations alone does not seem to provide a clear understanding about the level of variables among the respondents, mean percent was calculated applying this formula: ((mean score-minimum score) ÷ maximum score-minimum score))× 100. The differences in psychological wellbeing construct by demographic variables were analyzed using one-way Analysis of variance (ANOVA) or *t* test. Pearson correlation coefficient was applied to indicate the associations between psychological well-being and the psychological constructs. Moreover, hierarchical linear regression analysis with Enter method was applied to illustrate the variations in psychological wellbeing score on the basis of socio-demographic and psychological variables. A *P* value less than 0.05 was considered significant, a priori.

According to previous studies, demographic characteristics,^29^ perception on facts in daily living,^30^Healthy lifestyle,^31^ perceived social relationship^32^ and psychological constructs including self-efficacy,^33^hopefulness,^34^ life satisfaction,^35^ stress,^36,37^ happiness^21,38,39^ may influence on psychological wellbeing. A hierarchical multiple linear regression was performed in 6 blocks to assess the efficiency of the psychological constructs over the influence of other constructs. Predictors for the outcome variable were classified in 6 different blocks according to their natures: (1) Demographic characteristics block: number of family members, grade of birth, father’s job, mother’s job, father’s education, mother’s education, economic status, religious belief; (2) Perceptions on daily facts block: satisfaction with the parent’s job, willingness to modern live, satisfaction with family lifestyle, belief on chance in daily living, willingness to create happy time, willingness to watch TV, willingness to music; (3) Healthy lifestyle block: having the history of smoking, healthy diet, and physical activity; (4) Commitment to an action plan in daily life/take care about health block; (5) Perception on social relationship block: having favorite social relations, willingness to relation, have/had boyfriend; and (6) the psychological constructs block: self-efficacy, hopefulness, life satisfaction, stress, happiness.

Hierarchical multiple linear regressions were performed with psychological wellbeing and then perceived stress as outcomes. Considering the significant role of perceived stress found in the first model, we decided to run the hierarchical regression analysis once again to find the predictors of perceived stress.

## Results

Of the 300 students who were selected as the sample size, 289 were enrolled in the study, and 11 were excluded due to transfer to other areas. The socio-demographic characteristics of the participants along with their social relationship, having a healthy lifestyle, perceptions on daily facts and commitment to healthy action plan are displayed in Tables 1 and 2. About more than half of the students (51.6%) reported to have/had boyfriend. The majority had a good to fair social relationship, religious belief, economic situation and commitment to a healthy action plan (Table 2). Mean (standard deviation) for psychological well-being was 31.04 (15.33) (Table 3).

**Table 1:** Socio-demographic characteristics of the students and their associations with psychological wellbeing

**Socio-demographic characteristics**		**No. (%)**	**Mean**	***P*** ** value**
Number of family members	3	54 (18.7)	31.8	0.911
	4	186 (64.4)	30.7	
	≥5	49 (17)	31.1	
Grade of birth	1	146 (50.5)	30.3	0.642
	2	92 (31.8)	31.3	
	>3	51 (17.6)	32.5	
Father’s job	Self-employed	155 (53.6)	30.0	0.241
	Employee	134 (46.4)	32.1	
Mother’s job	Housewife	236 (81.7)	32.0	0.012
	Employed	53 (18.3)	26.4	
Father’s education	Elementary	71 (24.6)	29.1	0.473
	Diploma	120 (41.5)	31.8	
	University	98 (33.9)	31.4	
Mother’s education	Elementary	107 (37.0)	33.2	0.037
	Diploma	137 (47.4)	30.9	
	University	45 (15.6)	26.2	
Economic status	Moderate	83 (28.7)	33.3	0.021
	Good	185 (64.0)	29.3	
	Very good	21 (7.3)	37.3	
Religious belief	Low	32 (11.1)	36.7	0.001
	Moderate	128 (44.3)	33.0	
	High	107 (37.0)	27.0	
	>Very High	22 (7.6)	30.5	

Mean, standard deviations, mean percent, number of items, possible range and Cronbach α for the psychological constructs as well as their correlation coefficients with psychological wellbeing are presented in Table 3. The Cronbach α for the most of constructs was 0.7 and higher showing an acceptable to excellent internal consistency. The exception was hopefulness (α = 0.53). The level of scores for almost all variables was more than average, which means that the respondents acquired more than one half of the maximum possible score in almost all variables except for psychological wellbeing (mean percent = 36.9%). The lowest scores were found to be for stress (53.87%) and self-efficacy (64.37%). Applying Pearson correlation coefficient test, psychological well-being was found to have statistically significant positive correlations with all psychological constructs except for stress which was negatively associated with psychological well-being (r = -0.689) (Table 3).

As there is shown in Table 4, demographic characteristics of the respondents explained only 6.2% of the observed variance in psychological well-being (*P* < 0.001). In the subsequent blocks, significant effects were found on psychological well-being by Perceptions on daily facts (*P* < 0.001) (R^2^ = 0.152), healthy lifestyle (R^2^ = 0.212, *P* < 0.001), commitment to a healthy action plan/take care about health (R^2^ = 0.211, *P* < 0.001), perception on social relations (R^2^ = 0.279, *P* < 0.001) and the psychological variables (R^2^ = 0.595, *P* < 0.001).

In the final model (step 6), satisfaction with family lifestyle (β = 0.168, *P* < 0.001) and physical activity (β = 0.140, *P* < 0.001) were significant positive predictors for psychological well-being. Also, willingness to create happy times (β = -0.084, *P* < 0.040) and stress (β = -0.470, *P* < 0.001) were significant negative predictors for the outcome variable (R^2^ = 0.595, *P* < 0.001).

Considering the significant role of perceived stress in the final model, the hierarchical regression analysis was run once again to find the predictors of perceived stress (Table 5). In the first model, demographic characteristics explained only 3% of the observed variance in perceived stress (*P* < 0.05). In the second model (step 6), physical activity (β = -0.109, *P* < 0.019), have/had boyfriend (β = 0.237, *P* < 0.001), hopefulness (β = -0.130, *P* < 0.05) and happiness (-β = 0.387, *P* < 0.001) were significant predictors for perceived stress (R^2^ = 0.453, *P* < 0.001).

## Discussion

This study was conducted to investigate the determinants of psychological wellbeing among female adolescents in Tabriz, Iran. Understanding the psychological wellbeing status and identifying its influential factors among female adolescents may be helpful in addressing the issue through mental health promotion interventions.

In the current study, all the psychological constructs, except for perceived stress, were not significant predictors for mental health. In other words, at the presence of all underlying factors, perceived stress was the most significant negative predictor for psychological wellbeing among the female adolescents. This finding highlights the role of stress in determining psychological wellbeing of female adolescents. As there were several other variables in the present study that could potentially predict psychological wellbeing, it seems that perceived stress has overshadowed the influence of a majority of other variables. For instance, self-efficacy as a perception that determines an individual’s resilience to adversity and his/her vulnerability to depression,^40^ has been shown as an important influencing factor on human behavior and mental health.^41-43^ However, its association with psychological wellbeing in our study was not significant. We presume that such associations between the independent variables and outcome variable of our study are affected by perceived stress, considering the high impact of this variable in the final model of the present study as well as the high level of stress among Iranian female adolescents, as reported in the literature.^16,44^ Uncontrolled stress may have a detrimental impact on the physical and mental well-being of adolescent and may lead to various disorders such as depression.^36,37^ Based on our findings, training skills to create daily happy time events, like listening to music, dancing, and watching movie may increase self-efficacy and decrease the level of stress as steps toward achieving female adolescent’s psychological wellbeing in a country like Iran with specific religious socio-cultural limitations for women.

In the present study, economic status was one of the predictors for psychological wellbeing, in a way that the students with a better perception on the economic status of their families had a better status in their psychological wellbeing. This finding is consistent with those of Ho et al, who associated income disparities to psychological well-being among children.^29^ It has been well documented that poverty is negatively associated to physical and psychological well-being among adolescents and children.^38,39^ Mental disorders and stress among girls in developing countries are common and a public concern,^45^ as well. The World Health Organization (WHO) has focused on building evidence for the prevalence and causes of mental health, promoting health policies and enhancing competence of primary health care providers in such countries. But, this problem may be due to the cultural context and economic status of those countries, which urges the need for developing and applying comprehensive context-based social and community-based strategies with the hope to promote psychological well-being for female adolescents.

It seems that identifying of the students with low socioeconomic status as a vulnerable group during health promotion interventions in schools may be necessary to provide them with some facilities and supportive environments as well as financial aid with the hope to promote their level of mental health. Such students may be encouraged to participate more in the health promotion activities by providing them with gift cards, as a good incentive and financial assistance for mental health promotion.

One of the surprising results of our study was the negative contribution of willingness to create happy moments to psychological well-being, which means that the students with lower level of psychological wellbeing had more willingness to create happy moments. Moreover, our findings and previous studies have suggested links between happiness and psychological wellbeing.^17^ In other words, the students may feel the lack of happiness as a cause for their low level of psychological wellbeing, and therefore tend to find situations for more happy moments. Also, willingness to listening to music was one of the positive predictors of psychological well-being according to the findings of the present study. There is a large body of literature that has demonstrated the positive effects of music on improving the symptoms and reducing the severity of mental diseases like depression and schizophrenia.^46,47^ Schools could play a major role in promoting mental health programs and students’ mental health.^48^ Socio-cultural and political factors in Iran have resulted in the lack of hours for teaching music in schools which may be a contributing factor to the current mental health status of the students. Revising school curriculums to include music-related lessons like lawful anthemion, hymning, and chanting for female students in schools seems to be helpful in mental health promotion of the students. Also, creating supportive environments and activities such as designing and implementation of happy music-based programs in schools during official ceremonies and the recreation breaks and sports times may be useful, as well.

Having the history of smoking in previous year was a significant negative predictor for psychological well-being of the female adolescents in the present study. Smoking has strongly been associated to mental disorders. About one out of 3 adult smokers in the United States and Australia has been found to be with at least one disorder based on DSMIV and ICD-10,^49^ and as a comparison, only 15% of the current non-smoker adults were with a diagnosable mental disorder. A majority of adult smokers begin smoking in their adolescence years.^50^ Considering the increasing rate of beginning to smoke among women at younger ages,^51^ as well as the significance of peers and teachers as role models for the adolescents, establishing school-based campaigns on smoking prevention within which peer and teacher support are considered as the main strategies may be recommended. Such school-based campaigns may be also developed to the community and nation-wide levels with focus on strategies like awareness rising on the hazards of tobacco, developing life-skills training in kindergartens and elementary schools, and training parenting skills (like promoting family bonding and taught resistance skills).

## Limitations

There may be some limitations for our study. The first limitation is the self-report nature of the study which may has resulted in recall bias. Another limitation may be the correlational design of the research. Therefore, causal inferences are warranted. We had limitations in asking questions on some culturally sensitive questions, like alcohol consumption and having sexual relationships. We propose further researches with more focus on the associations of such factors with psychological wellbeing among female adolescents.

## Conclusion

Our finding highlights the role of perceived stress in determining psychological wellbeing of female adolescents. As there were several other determinants in the present study that could potentially predict psychological wellbeing, it seems that perceived stress has overshadowed the influence of a majority of the other factors. Further longitudinal and filed trial researches are suggested to investigate these associations among female adolescents in different communities. School health policymakers and school nurses should consider these findings while designing mental health promotion policies and programs for adolescents, particularly when addressing the issues that may be associated to the role of families, friends, peers and communities in maintaining and promoting adolescents’ mental health.

## Ethical approval

All procedures performed in studies involving human participants were in accordance with the ethical standards of the institutional and/or national research committee and with the 1964 Helsinki Declaration and its later amendments or comparable ethical standards. Ethical approval was provided by Student Research Committee in Tabriz University of Medical Sciences. Also, Informed consent was obtained from all individual participants included in the study.

## Competing interests

The authors declare that they have no competing interests.

## Authors’ contributions

HN and HH conceptualized and designed the study, assisted in data collection, carried out the analyses, made substantial contributions to interpretation of data and drafted the initial manuscript, reviewed and revised the final manuscript. Both the authors read and approved the final manuscript.

## Acknowledgements

Authors thank all the students participated in the study.

**Table 2:** Perception of the students on some facts in daily living and their associations with psychological wellbeing

**Variables**		**No. (%)**	**Mean**	***P*** ** value**
Satisfaction with the parent’s job	Yes	238 (82.4)	30.0	0.021
	No	51 (17.6)	35.4	
Satisfaction with family lifestyle	Yes	221 (76.5)	28.2	0.004
	No	68 (23.5)	40.1	
Belief on chance in daily living	Yes	198 (68.5)	31.9	0.156
	No	91 (31.5)	29.1	
Willingness to create happy times	Yes	277 (95.8)	30.7	0.142
	No	2 (4.2)	37.3	
Willingness to watch TV	Yes	192 (66.4)	31.5	0.432
	No	97 (33.6)	30.0	
Willingness to music	Yes	230 (79.6)	31.7	0.132
	No	59 (20.4)	28.4	
Take care about health	Yes	234 (81.0)	29.4	.002
	No	55 (19.0)	37.6	
Commitment to an action plan in daily life	Yes	210 (72.7)	29.1	0.003
	No	79 (27.3)	36.0	
Social relationship				
Having favorite social relationship	No	157 (54.3)	36.2	0.003
	Yes	132 (45.7)	27.3	
Willingness to social relation	No	58 (20.1)	26.6	0.015
	Yes	231 (79.9)	32.1	
Have/had boyfriend	Yes	149 (51.6)	35.3	0.001
	No	140 (48.4)	26.4	
Healthy lifestyle				
Having the history of smoking	Yes	11 (3.8)	40.2	0.048
	No	278 (96.2)	30.6	
Healthy diet	Yes	100 (34.6)	27.2	0.009
	No	189 (65.4)	33.0	
Physical activity	Yes	48 (16.6)	27.0	0.041
	No	241 (83.4)	31.8	

**Table 3:** Descriptive statistics for the psychological variables and their correlation coefficients with psychological wellbeing

**Major variables**	**Mean (SD)**	**Mean percent**	**Number of items**	**Possible range**	**Cronbach α**	**r**
Self-efficacy	58.37 (10.10)	64.37	17	17-85	0.81	0.424**
Hopefulness	33.60 (3.88)	66.66	12	12-48	0.53	0.406**
Life satisfaction	20.75 (6.48)	90.47	5	5-35	0.80	0.386*
Stress	23.65 (5.75)	53.87	10	0-40	0.69	-0.689**
Happiness	113.25 (19.50)	72.11	29	29-174	0.85	0.536**
Psychological well-being	31.04 (15.33)	36.95	28	0-84	0.92	1

*P value is significant at *P* < 0.01; **P value is significant at *P* < 0.001.

**Table 4:** Hierarchical linear regression analysis to predict psychological well-being

Step/variable	**Step 1** **β (** ***P*** ** value)**	**Step 2** **β (** ***P*** ** value)**	**Step 3** **β (** ***P*** ** value)**	**Step 4** **β (** ***P*** ** value)**	**Step 5** **β (** ***P*** ** value)**	**Step 6** **β (** ***P*** ** value)**
Outcome variable: Psychological Wellbeing						
1) Number of family members	-0.059 (0.331)	-0.070 (0.236)	-0.072 (0.215)	-0.072 (0.215)	-0.046 (0.410)	-0.021 (0.627)
Grade of birth	-0.001 (0.987)	0.001 (0.984)	0.015 (0.803)	0.014 (0.816)	0.013 (0.815)	0.050 (0.250)
Father’s job	0.040 (0.569)	0.073 (0.280)	0.098 (0.135)	0.099 (0.132)	0.080 (0.202)	0.000 (0.995)
Mother’s job	-0.107 (0.112)	-0.094 (0.152)	-0.080 (0.209)	-0.078 (0.221)	-0.082 (0.180)	-0.073 (0.111)
Father’s education	0.142 (0.071)	0.112 (0.140)	0.112 (0.127)	0.106 (0.152)	0.077 (0.278)	0.083 (0.120)
Mother’s education	**0.202 (0.012)**	**0.186 (0.016)**	**0.209 (0.005)**	**0.206 (0.006)**	**0.166 (0.022)**	0.105 (0.055)
Economic status	0.010 (0.869)	0.013 (0.829)	0.003 (0.952)	0.039 (0.277)	0.047 (0.113)	**0.084 (0.041)**
Religious belief	**-0.180 (0.002)**	-0.093 (0.105)	-0.087 (0.115)	-0.079 (0.160)	-0.033 (0.552)	-0.031 (0.462)
2) Satisfaction with the parent’s job		-0.103 (0.085)	**-0.116 (0.045)**	**-0.115 (0.048)**	-0.063 (0.265)	-0.019 (0.652)
Satisfaction with family lifestyle		**0.270 (0.000)**	**0.242 (0.000)**	**0.238 (0.000)**	**0.201 (0.000)**	**0.168 (0.000)**
Belief on chance in daily living		0.066 (0.233)	0.026 (0.634)	0.024 (0.667)	0.015 (0.773)	-0.037 (0.350)
Willingness to create happy times		**-0.112 (0.050)**	-0.103 (0.063)	-0.098 (0.077)	**-0.106 (0.047)**	**-0.084 (0.040)**
Willingness to watch TV		0.038 (0.492)	0.031 (0.566)	0.026 (0.621)	0.057 (0.268)	0.032 (0.417)
Willingness to listening to music		0.077 (0.170)	0.066 (0.231)	0.067 (0.220)	0.033 (0.535)	0.049 (0.216)
3) Having history of smoking			-0.098 (0.071)	-0.094 (0.084)	-0.084 (0.109)	**-0.072 (0.036)**
Healthy diet			**0.148 (0.010)**	**0.143 (0.013)**	**0.145 (0.009)**	0.072 (0.094)
Physical activity			**0.124 (0.034)**	**0.120 (0.041)**	**0.123 (0.028)**	**0.140 (0.001)**
4) Commitment to a healthy action plan			-0.099 (0.068)	-0.097 (0.075)	-0.084 (0.109)	-0.019 (0.641)
Take care about health				0.042 (0.463)	0.015 (0.786)	0.078 (0.076)
5) Having favorite social relations					**0.176 (0.001)**	0.003 (0.946)
Willingness to relation					**0.136 (0.010)**	0.040 (0.319)
Have/had boyfriend					**0.181 (0.001)**	0.014 (0.741)
6) Self-efficacy						0.082 (0.151)
Hopefulness						0.107 (0.051)
Life satisfaction						0.026 (0.609)
Stress						**-0.470 (0.000)**
Happiness						0.112 (0.064)
R^2^	0.062	0.152	0.212	0.211	0.279	0.595
*P* value	<0.001	<0.001	<0.001	<0.001	<0.001	<0.001

**Table 5:** Hierarchical linear regression analysis to predict perceived stress

Step/variable	**Step 1** **β (** ***P*** ** value)**	**Step 2** **β (** ***P*** ** value)**	**Step 3** **β (** ***P*** ** value)**	**Step 4** **β (** ***P*** ** value)**	**Step 5** **β (** ***P*** ** value)**	**Step 6** **β (** ***P*** ** value)**
Outcome variable: Perceived stress						
1) Number of family members	-0.035 (0.573)	-0.015 (0.805)	-0.024 (0.691)	-0.034 (0.577)	-0.009 (0.880)	0.001 (0.979)
Grade of birth	-0.052 (0.432)	-0.028 (0.654)	0.028 (0.659)	0.035 (0.574)	-0.041 (0.484)	-0.027 (0.591)
Father’s job	0.117 (0.097)	0.118 (0.085)	0.121 (0.075)	0.128 (0.060)	0.118 (0.069)	0.093 (0.091)
Mother’s job	-0.029 (0.676)	-0.003 (0.969)	0.008 (0.897)	-0.015 (0.823)	-0.006 (0.926)	0.009 (0.860)
Father’s education	0.083 (0.295)	0.024 (0.758)	0.031 (0.689)	0.026 (0.733)	0.007 (0.922)	0.040 (0.523)
Mother’s education	-0.134 (0.101)	-0.094 (0.227)	**-**0.100 (0.195)	-0.109 (0.160)	-0.077 (0.299)	-0.043 (0.501)
Economic status	-0.073 (0.222)	-0.041 (0.488)	-0.040 (0.499)	-0.039 (0.508)	-0.021 (0.708)	0.027 (0.577**)**
Religious belief	**-0.138 (0.020)**	-0.040 (0.494)	-0.030 (0.605)	-0.018 (0.759)	0.024 (0.663)	-0.047 (0.331)
2) Satisfaction with the parent’s job		-0.062 (0.309)	-0.083 (0.168)	-0.085 (0.159)	-0.030 (0.598)	0.005 (0.914)
Satisfaction with family lifestyle		**-0.309 (0.000)**	**0.296 (0.000)**	**-0.269 (0.000)**	**-0.184 (0.002)**	-0.026 (0.633)
Belief on chance in daily living		**0.143 (0.012)**	**0.113 (0.047)**	0.111 (0.051)	0.096 (0.075)	0.080 (0.081)
Willingness to create happy times		-0.030 (0.600)	-0.032 (0.570)	-0.025 (0.664)	-0.032 (0.559)	-0.001 (0.975)
Willingness to watch TV		-0.006 (0.920)	0.004 (0.935)	-0.002 (0.970)	0.026 (0.618)	0.003 (0.942)
Willingness to listening to music		0.039 (0.494)	0.027 (0.634)	0.036 (0.528)	-0.005 (0.920)	0.030 (0.512)
3) Having history of smoking			-0.056 (0.321)	-0.047 (0.406)	-0.035 (0.516)	0.006 (0.905)
Healthy diet			-0.038 (0.504)	-0.004 (0.942)	-0.001 (0.986)	-0.030 (0.546)
Physical activity			**-0.174 (0.002)**	**-0.172 (0.003)**	**-0.156 (0.004)**	**-0.109 (0.019)**
4) Commitment to a healthy action plan				-0.073 (0.221)	-0.052 (0.360)	0.017 (0.742)
Take care about health				-0.070 (0.238)	-0.082 (0.147)	-0.014 (0.782)
5) Having favorite social relations					**-0.139 (0.013)**	0.038 (0.448)
Willingness to relation					**0.113 (0.037)**	0.051 (0.275)
Have/had boyfriend					**0.271 (0.000)**	**0.237 (0.000)**
6) Self-efficacy						-0.076 (0.253)
Hopefulness						**-0.130 (0.040)**
Life satisfaction						-0.094 (0.115)
Happiness						**-0.387 (0.000)**
*R* ^ 2 ^	0.030	0.128	0.154	0.157	0.245	0.453
*P* value	0.036	<0.001	<0.001	<0.001	<0.001	<0.001
